# Self-Injurious Behavior in Adolescents

**DOI:** 10.1371/journal.pmed.1000240

**Published:** 2010-05-25

**Authors:** Janis Whitlock

**Affiliations:** Family Life Development Center, Cornell University, Ithaca, New York, United States of America

## Abstract

Janis Whitlock discusses the epidemiology and and care of adolescents undertaking nonsuicidal self-injury, also called “deliberate self-harm.”

## Introduction

What constitutes non-suicidal self-injury (NSSI) is a matter of some debate, but its growing presence in mainstream and popular media as well as the growing number of anecdotal reports by physicians, therapists, and junior and senior high school counselors suggest that it may be, as some have called it, “the next teen disorder” [1]. Referred to in the literature and media as “self-injurious behavior,” “self-injury,” “self-harm,” “self-mutilation,” or “cutting,” self-injury is typically defined as the deliberate, self-inflicted destruction of body tissue without suicidal intent and for purposes not socially sanctioned [2]. Although most often not a suicidal gesture, it is statistically associated with suicide and can result in unanticipated severe harm or fatality [3],[4],[5].

## What Do We Know about NSSI Prevalence and Characteristics in Adolescents?

Although study of NSSI in adolescence is relatively new, empirical advances in NSSI research over the past several years have resulted in a solid foundation of knowledge about basic epidemiological parameters. Many normally developing youth practice what is typically referred to as *common* NSSI [6]. This form of self-injury includes NSSI that is (a) compulsive (ritualistic and rarely premeditated such as hair pulling or trichotillomania), (b) episodic (every so often and with no identification as someone who self-injures), and (c) repetitive (performed on a regular basis and with ego identification as someone who self-injures). Common NSSI can be mild, moderate, or severe depending on the lethality of the injuries. Although *common* NSSI can and does co-occur with other DSM classifiable mental illnesses, such as depression or anxiety, it is also increasingly evident that it presents independently of other mental illness [7].

In general, U.S. studies tend to find that lifetime prevalence of common NSSI ranges from 12% to 37.2% in secondary school populations [8] and 12% to 20% [7],[9] in late adolescent and young adult populations. NSSI scholarship consistently shows an average age of onset between 11 and 15 y [8],[9],[10],[11],[12] with a normally distributed age of onset ranging from about 10–24 [9]. Of all youth reporting any NSSI, over three quarters report repeat NSSI (>1 episode) [9] and an estimated 6%–7% of adolescents report current repetitive NSSI (NSSI in the past year) [7],[8],[9]. Overall, about a quarter of all adolescents and young adults with NSSI history report practicing NSSI only once in their lives [9],[13], but since even a single NSSI episode is significantly correlated with a history of abuse and comorbid conditions such as suicidality and psychiatric distress, there may be a group of adolescents in which a single incident of NSSI serves as a risk indicator for other risk behaviors or pathology [9]. Duration of NSSI is understudied, but available evidence suggests that among individuals with a history of repeat NSSI, the majority (79.8%) reported stopping NSSI within 5 y of starting and 40% reported stopping within 1 y of starting [9].

NSSI differs from culturally sanctioned self-injury, such as piercing or tattooing, by intention rather than form as well as by injurious agent (piercing and tattooing are most commonly performed by someone other than oneself, while the reverse is usually true for NSSI). Although most often associated with the term “cutting,” the most common forms among youth include scratching, cutting, punching, or banging objects with the conscious intention of self-injury; punching or banging oneself; biting, ripping, or tearing the skin; carving on the self; and burning [9],[13],[14],[15],[16]. Where on the body one injures may be important as well. Injuries inflicted on the face, eyes, neck in the jugular region, breast, or genitals, for instance, may be clinically indicative of greater psychological disturbance than when injuries are inflicted elsewhere [17],[18]. The majority of young people reporting repeat self-injury also report using multiple methods and multiple body locations [9].

Most studies show females slightly more likely to practice NSSI than males (unpublished data) [9],[19]. Recent work suggests that there may be different self-injury groups or “classes,” one of which consists largely of men who use self-injury forms that can be described as “self-battery” and/or who practice NSSI in social settings [20]. Findings with regard to race and NSSI are mixed, with some studies suggesting that it may be more common among Caucasians [21] and others showing similarly high rates in minority samples [9],[22]. There is also evidence linking NSSI to sexual orientation such that incidence of NSSI is slightly elevated among those who report exclusive homosexual attraction and some same-sex attraction, and it is very elevated among individuals with bisexual and questioning sexual orientation status (unpublished data) [9].

Although empirical attention devoted to NSSI varies dramatically around the world, it is clear that NSSI is globally present and prevalent. The U.K., for example, has dedicated national resources to investigation and reduction of “self-harm” among youth [18], and scholars from both Canada and Europe [23],[24] have documented alarmingly high rates of self-harm in their countries. Although most widely investigated in industrialized regions such as Europe, North America, Australia, and New Zealand, NSSI also occurs with some regularity in other industrialized and non-industrialized countries as well [21],[22],[25]. However, comparing rates and characteristics of NSSI internationally is complicated by the fact that many measures of NSSI outside of the U.S. (most commonly referred to as “self-harm”) include behaviors undertaken with suicidal intent and may also capture socially sanctioned self-injurious behaviors, such as those used as part of religious or ritualistic practices [25].

## Why Do Youth Self-Injure?

In general, reasons for self-injuring break down into three general categories: psychological, social, and biological. Of these, psychological functions are most commonly cited and center around reducing psychological pain, expressing and alleviating psychological distress, and refocusing one's attention away from negative stimulus [12],[17],[26]. Much less common but sometimes cited are reasons such as “so someone would pay attention” and “to get a rush or surge of energy.” Both underscore the role of both social and biological roles in maintaining NSSI. Social function models point to the importance of viewing NSSI as a behavior undertaken to fulfill multiple functions simultaneously, most of which are intrapersonal (emotion regulation) but some of which are fundamentally interpersonal in nature. In addition to being identified as factors that predispose or place at-risk adolescents who ultimately adopt NSSI as a release for negative emotion [27],[28], research finds interpersonal factors also make significant contributions to NSSI maintenance [12],[27],[28]. Biological models of function tend to focus primarily on the role of NSSI in regulation of endogenous opioids. The homeostasis model of NSSI, for example, suggests that individuals who self-injure may have chronically lower than normal levels of endogenous opioids. In this model, NSSI is fundamentally remedial—it represents an attempt to restore opioids to normal levels. Low levels of opioids may result from a history of abuse, trauma, or neglect or may be biologically endowed through other processes [29]. These models are very helpful in deepening understanding about how and why some individuals perceive that they are dependent on NSSI behavior for emotion regulation.

Identifying unique antecedents to NSSI is more difficult since it shares with many adolescent risk behaviors predisposing factors such as emotion dysregulation, self-derogation, childhood adversity, and comorbid or antecedent psychiatric disorders [30]. In clinical populations, self-injury is strongly linked to childhood abuse, especially childhood sexual abuse [27],[31]. Self-injury is also linked to eating disorders, substance abuse, post-traumatic stress disorder, borderline personality disorder, depression, and anxiety disorders [27]. While much of this research reflects comorbidity in clinical populations, more recent studies of these relationships in community populations of youth document similar patterns, though at significantly lower levels of association [7],[9],[32]. Indeed, one study found that 44% of respondents with current NSSI behavior evidenced no existing comorbid clinical conditions [7].

## What Is the Relationship between NSSI and Suicide?

That NSSI and suicide behaviors are related is well documented [3]–[5], but the nature of its relationship remains somewhat ambiguous. Most NSSI treatment specialists and scholars agree that in the vast majority of cases NSSI is utilized to temporarily alleviate distress rather than to signal the intention to end one's life [17],[25],[33]. Indeed, some see it as a means of avoiding suicide [34],[35]. Thus, in its relation to suicide, NSSI possesses an ambiguous, seemingly paradoxical, status as both a temporarily functional means of sustaining life by reducing and regulating strong negative emotion while simultaneously serving as a potential harbinger for suicidal intent and attempts. This dual status suggests that efforts to discern variations in motivation and intent may be the most productive means of generating information useful in tailoring treatment guidelines, materials, and services. While Walsh [17] has argued that NSSI and suicide are entirely distinct psychological and behavioral phenomenon, Joiner theorizes that some suicidal individuals acquire the capacity to engage in high lethality behavior (i.e., suicide) by engaging in increasingly severe NSSI over time [36]. Assuming that suicide behavior is a *consequence* of NSSI behavior assumes a temporal relationship that has yet to be documented. If this assumption proves true, then the data would suggest that for some NSSI serves as a harbinger of distress that, if left unmitigated, may lead some individuals to consider or attempt suicide later.

## Is NSSI Contagious?

It is widely assumed that NSSI is contagious, although lack of empirical data necessarily limits our capacity to test this assumption. Nevertheless, studies of contagion among adolescents in clinical settings demonstrate the tendency for NSSI to spread in a population [37]–[39] and the presence of self-injury in media, such as in music, movies, and newspapers, has increased dramatically in the past several years [40]. The Internet, as well, has proven to be a popular avenue for the gathering of individuals who practice NSSI [41]. Studies of the social contexts of behavior consistently show that positive and negative behaviors are socially patterned and often clustered [42] and that the primary mechanism of spread tends to be through (a) the shaping of norms, (b) providing social reinforcement of behaviors, (c) providing (or limiting) opportunities to engage in the behavior, and (d) facilitating or inhibiting the antecedents for the behavior [42]. Considered together, these mechanisms provide a useful framework for understanding how self-injury might spread in community populations of youth and point to the need for prevention and intervention approaches that address each of these areas.

## How Is NSSI Best Treated?

Although NSSI treatment specialists can offer advice based on experience, few studies that actually test treatment strategies have been conducted. In a systematic review of 23 randomized controlled trials related to Deliberate Self Harm (a U.K.-based term that includes NSSI and suicide-related behavior), reviewers concluded that the most promising approaches include problem-solving therapy, provision of emergency service contact information, long-term psychological therapy, and depot flupenthixol (for those with repeat self-harm experience). They caution, however, that current knowledge is insufficient and more trials are sorely needed [43]. In a systematic review of NSSI-specific treatment strategies, Muehlenkamp concludes that approaches utilizing largely cognitive-behavioral therapy (CBT) may prove most efficacious in NSSI treatment [44]. Because of the time-limited and structured coping skill-building nature of the technique, she specifically identifies problem-solving therapy and dialectical behavioral therapy as the most promising CBT-based candidates but suggests that while both may be efficacious under the right treatment conditions, neither has emerged as efficacious in the limited study available. Although dialectical behavior therapy has been used with significant success in borderline personality disordered patients with suicide and NSSI as well [46], there is significant need for well-designed and rigorous trials of NSSI treatment strategies among community populations.

## How Do We Detect NSSI?

Although common among adolescents, NSSI is often undetected. Medical providers are uniquely positioned to assess for NSSI behavior during intake assessments and during examination since wounds or scars may be visible. Arms, fists, and forearms opposite the dominant hand are common areas for injury. However, evidence of self-injurious acts can and do appear anywhere on the body. Other signs include inappropriate dress for season (consistently wearing long sleeves or pants in summer), constant use of wrist bands/coverings, unwillingness to participate in events/activities that require less body coverage (such as swimming or gym class), and frequent bandages and odd/unexplainable paraphernalia (e.g., razor blades or other implements that could be used to cut or pound). It is important that questions about the marks be non-threatening and emotionally neutral. Treatment veteran Barent Walsh indicates that he has the most success making patients comfortable and gleaning clinically useful information by demonstrating “respectful curiosity” toward individuals with NSSI history [17].

If NSSI is detected, health professionals should investigate and address:

- Immediate risk of infection: Open wounds should be assessed for likelihood of infection. Even in cases where wounds are healed, a discussion of how to care for wounds is warranted. This is particularly important since a significant number of those with NSSI experience indicate inflicting wounds of unintended severity [9],[17].
- NSSI severity: In general, lifetime frequency of NSSI in combination with the number of methods used and the likelihood that the methods used will cause severe tissue damage (i.e., cutting, burning, bone breaking, etc.) is directly and positively correlated with risk of other adverse outcomes, such as suicide-related behaviors and global psychological distress. High-severity cases (high lifetime frequency, injury in the past 6 mo, use of forms likely to inflict high tissue damage, and/or use of multiple forms) warrant thorough assessment of existing therapeutic support and referral if found inadequate or lacking.
- Extent of informal and formal support system: Has the patient disclosed injury to anyone, and if so, how supportive are those who know? Does the patient currently receive therapy in which presence of NSSI has been disclosed? If not, referral is warranted—particularly for high-severity cases.
- Presence of comorbid mental health conditions, such as disordered eating, depression, anxiety, borderline personality disorder, and generalized psychological distress. Presence of one or more of these conditions in NSSI patients is common and may heighten risk of suicide [3],[19],[46].
- Suicide assessment: Although NSSI is not a suicidal gesture, it can indicate the presence of suicidal thoughts and feelings and should trigger suicide assessment in individuals who have self-injured in the previous year. A variety of assessment tools are available to do this, including but not limited to the SI-IAT [47] and the Beck Suicide Intent Scale [48].

## Summary

NSSI is a common practice among adolescents, and medical providers are uniquely positioned to detect its presence, to assess its lethality, and to assist patients in caring for wounds and in seeking psychological treatment. NSSI assessment should be standard practice in medical settings. Randomized control trials of effective treatment and prevention strategies are warranted. Because NSSI research is nascent, unanswered research questions abound. Those most pressing for clinicians and allied medical health professionals include (a) discerning individuals with NSSI history at elevated risk for suicide from those not at elevated risk, (b) effective treatment regimes, (c) effective prevention strategies in school and community settings, and (d) assessment and referral protocols likely to result in effective treatment and abatement of NSSI behavior.

**Five Key Studies in the Field**

1. Ross S, Heath N (2002) A study of the frequency of self-mutilation in a community sample of adolescents. J Youth Adolesc 31: 66–77.

This is one of the first descriptive studies of NSSI in a high school sample of adolescents. It paved the way for study of NSSI in community populations by documenting a high prevalence rate and providing novel descriptive details [24].

2. Nock MK, Prinstein MJ (2004) A functional approach to the assessment of self-mutilative behavior. J Consult Clin Psychol 72: 885–890.

This is the first study to document a functional model of NSSI that moved beyond the pejorative manipulation function and provided empirical support for a multi-functional conceptualization of NSSI in adolescents [12].

3. Whitlock J, Eckenrode J, Silverman D (2006) Self-injurious behaviors in a college population. Pediatrics 117: 1939–1948.

This was the first large-scale epidemiological study to document the phenomena of NSSI in college students and to provide detailed epidemiological portraits of the phenomenon [9].

4. Muehlenkamp J, Gutierrez PM (2007) Risk for suicide attempts among adolescents who engage in non-suicidal self-injury. Arch Suicide Res 11: 69–82.

This was among the very first empirical papers to document the distinctions between NSSI and suicide beyond the intent of the behavior, and did so within a community sample of high school students, expanding research on NSSI to nonclinical settings [4].

5. Rossow I, Ystgaard M, Hawton K, Madge N, van Heeringen K, et al. (2007) Cross-national comparisons of the association between alcohol consumption and deliberate self-harm in adolescents. Suicide Life Threat Behav 37: 605–615.

This was the first large-scale international study of NSSI prevalence (called “deliberate self harm” in Europe). It also paved the way for looking at the relationship between NSSI and common adolescent risk behaviors such as alcohol use [25].
